# Exploring brain glutathione and peripheral blood markers in posttraumatic stress disorder: a combined [1H]MRS and peripheral blood study

**DOI:** 10.3389/fpsyt.2023.1195012

**Published:** 2023-06-02

**Authors:** Sarah E. Watling, Shawn G. Rhind, Jerry Warsh, Duncan Green, Tina McCluskey, Junchao Tong, Peter Truong, Sofia Chavez, J. Don Richardson, Stephen J. Kish, Isabelle Boileau

**Affiliations:** ^1^Institute of Medical Sciences, University of Toronto, Toronto, ON, Canada; ^2^Brain Health Imaging Centre, Centre for Addiction and Mental Health, Toronto, ON, Canada; ^3^Faculty of Kinesiology & Physical Education, University of Toronto, Toronto, ON, Canada; ^4^Defence Research and Development Canada, Toronto Research Centre, Toronto, ON, Canada; ^5^Campbell Mental Health Research Institute, Centre for Addiction and Mental Health, Toronto, ON, Canada; ^6^Department of Psychiatry, University of Toronto, Toronto, ON, Canada; ^7^Department of Pharmacology and Toxicology, University of Toronto, Toronto, ON, Canada; ^8^The MacDonald Franklin Operational Stress Injury (OSI) Research Centre, Lawson Health Research Institute, London, ON, Canada; ^9^Department of Psychiatry, Western University, London, ON, Canada; ^10^Department of Psychiatry and Behavioural Neurosciences, McMaster University, Hamilton, ON, Canada; ^11^St. Joseph's London Operational Stress Injury (OSI), Parkwood Institute, St. Joseph's Health Care, London, ON, Canada

**Keywords:** magnetic resonance spectroscopy, glutathione, metalloproteinase (MMP), myeloperoxidase (MPO), psychiatric disorder, posttraumatic stress disorder (PTSD)

## Abstract

**Introduction:**

Oxidative stress has been implicated in psychiatric disorders, including posttraumatic stress disorder (PTSD). Currently, the status of glutathione (GSH), the brain's most abundant antioxidant, in PTSD remains uncertain. Therefore, the current study investigated brain concentrations of GSH and peripheral concentrations of blood markers in individuals with PTSD vs. Healthy Controls (HC).

**Methods:**

GSH spectra was acquired in the anterior cingulate cortex (ACC) and dorsolateral prefrontal cortex (DLPFC) using MEGA-PRESS, a J-difference-editing acquisition method. Peripheral blood samples were analyzed for concentrations of metalloproteinase (MMP)-9, tissue inhibitors of MMP (TIMP)-1,2, and myeloperoxidase (MPO).

**Results:**

There was no difference in GSH between PTSD and HC in the ACC (*n* = 30 PTSD, *n* = 20 HC) or DLPFC (*n* = 14 PTSD, *n* = 18 HC). There were no group differences between peripheral blood markers (*P* > 0.3) except for (non-significantly) lower TIMP-2 in PTSD. Additionally, TIMP-2 and GSH in the ACC were positively related in those with PTSD. Finally, MPO and MMP-9 were negatively associated with duration of PTSD.

**Conclusions:**

We do not report altered GSH concentrations in the ACC or DLPFC in PTSD, however, systemic MMPs and MPO might be implicated in central processes and progression of PTSD. Future research should investigate these relationships in larger sample sizes.

## 1. Introduction

There is a need to develop effective medication for posttraumatic stress disorder (PTSD), as currently, < 30% of people diagnosed with PTSD will achieve remission (1). Employing brain imaging to understand the underlying neurobiology of PTSD can identify therapeutic targets. Magnetic Resonance Spectroscopy (MRS) is a valuable imaging modality that enables the *in vivo* measurement of brain-based metabolites. Indeed, MRS has been employed in PTSD to measure selected brain metabolites; to date the literature reports decreases in N-acetylaspartate (NAA) a marker of neuronal integrity and density, and mixed or null findings of glutamate (Glu), γ-aminobutyric acid (GABA), myo-inositol (mI), and choline (Cho) (2). Since NAA is a mitochondrial metabolite, decreased NAA could reflect damaged mitochondria in cerebral tissue (3). Oxidative stress is a primary driver of mitochondrial dysfunction/damage (4). Interestingly, the status of glutathione (GSH), the brain main's antioxidant in PTSD remains uncertain, as to our knowledge, the one study (5), investigating GSH in PTSD employed an MRS sequence that is not validated to measure GSH in the brain [see Rae and Williams (6) and discussion].

It is not surprising that oxidative stress and redox biology have been implicated in PTSD (7, 8), considering that the brain in particular is susceptible to oxidative stress related damage due to the central organ's high lipid content and metabolic rate (7). Notably, relevant features of PTSD, including dysregulated hypothalamic pituitary adrenal (HPA) axis (9), sleep disturbances (10), metabolic syndromes (11), neurodegeneration (12), inflammation (13), and brain atrophy (14) are also associated with oxidative stress (15). Preclinical research investigating oxidative stress in animal models of PTSD has reported decreased antioxidants (e.g., GSH) or increased free radical by-products [e.g., malondialdehyde (MDA)] and related enzymes in various regions of the brain such as the hippocampus (16–19), amygdala (20, 21), and prefrontal cortex (PFC) (20, 22, 23). Human post-mortem brain analysis has also reported alterations in genes related to oxidative stress in the dorsolateral PFC (DLPFC) of patients with PTSD (24). GSH plays an important role in protecting the central nervous system from oxidative stress related damage, where it serves as a co-factor for antioxidant enzymes including glutathione peroxidase, which functions to detoxify peroxides, as well as glutathione-s-transferase, which functions to reverse oxidized protein residues (25), and is, therefore, a useful molecule to investigate to understand the role oxidative stress might have in PTSD.

Also of interest, are various molecules circulating in peripheral blood that might relate to central markers of oxidative stress. Several lines of inquiry have investigated concentrations of common inflammatory mediators, including interleukin (IL)-1β, IL-6, and tumor necrosis factor (TNF)- α, with mixed results reported (26). An emerging line of interest (not yet investigated in humans living with PTSD), has begun to explore matrix metalloproteinases (MMPs) and their tissue inhibitors (tissue inhibitor of metalloproteinases (TIMPs) in psychiatric disorders (27). MMPs are extracellular matrix (ECM) proteins that help degrade ECM molecules and release growth factors. MMP-9 (28–30) and TIMP-2 (31) have been implicated in preclinical models of PTSD and its clinical features including learned fear. Also of interest is myeloperoxidase (MPO), a peroxidase enzyme that can catalyze the formation of ROS. MPO has also been implicated in PTSD (32), as well as major depressive disorder (MDD) (33). While these proteins are not by-products of oxidative stress or antioxidants, they can be activated by oxidative stress and correlate with TBARS (by-product of lipid peroxidation) (34, 35). To date, the relationship between these peripheral markers and brain GSH has not been explored. Therefore, the current study employed MEGA-PRESS (optimized for GSH) to quantify brain GSH in the ACC and DLPFC [to replicate previous research (5)] in people with PTSD and healthy controls. The investigation of brain GSH was complemented by the measurement of plasma levels of MMP-9, MPO, TIMP-1&2. Our primary aim was to investigate group differences in brain GSH and peripheral blood markers; secondarily, we aimed to explore relationships between central and peripheral markers and features/symptoms related to PTSD.

## 2. Materials and methods

### 2.1. Participants

This study was performed from September 2017 to October 2022 at the Center for Addiction and Mental Health (CAMH, Toronto, ON). After receiving approval for this study from the CAMH Research Ethics Board, research participants were recruited from the Greater Toronto Area and relevant communities**/**clinics (including Parkwood Operational Stress Injury Clinic, London ON) using posted and online advertisements and brochures. After providing written informed consent, research participants completed a comprehensive medical and psychiatric screening assessment (using the structured clinical interview for DSM (SCID)-IV/5) (36) and questionnaires assessing mood [Generalized Anxiety Disorder (GAD)-7 (37), Patient Health Questionnaire (PHQ)-9 (38), Beck Depression Inventory (BDI) (39)], PTSD symptoms [PTSD symptom scale (PSS) (40) and PTSD Checklist (PCL) (41)], and the traumatic life events questionnaire (42) at CAMH. Research staff also collected urine samples to screen for drug use, medication and pregnancy in female participants. Volunteers were eligible to participate if they were 17 years old or older, physically healthy, and had no current or previous DSM Axis I diagnosis except co-morbid mood disorder with PTSD (PTSD group only). PTSD participants were included if they met criteria for PTSD based on the Structured Clinical Interview for DSM-IV-5. Cannabis and medication use was not exclusionary in the PTSD group as long as participants did not meet criteria for current substance use disorder (according to DSM-IV/5 criteria). Nicotine dependence was not exclusionary in both study groups.

### 2.2. MRI session

On magnetic resonance imaging (MRI) scan day, urine toxicology (BTNX Inc. Pickering, Canada), breath alcohol and expired carbon monoxide measurements were taken to assess recent alcohol and smoking. Additionally, a urine sample was collected to detect substance use and medications (and to confirm that female participants were not pregnant). MRI scans took place in a 3T GE Discovery scanner (GE Healthcare; SW: DV26 201) in the Brain Health Imaging Center at CAMH for ~1.5 h. To minimize head movement, each participant was positioned at the center of the eight-channel head coil with soft padding around the head. Magnet homogeneity was adjusted using the manufacture automated shimming routine. High resolution SagT1-weighted BRAVO images were obtained for each participant [echo time (TE) = 3.016 ms; recovery time (TR) = 6.768 ms; field of view (FOV) = 256 × 256; scan time = 4:42 (min:sec)].

### 2.3. MRS data acquisition and analysis

**Figure 1:**
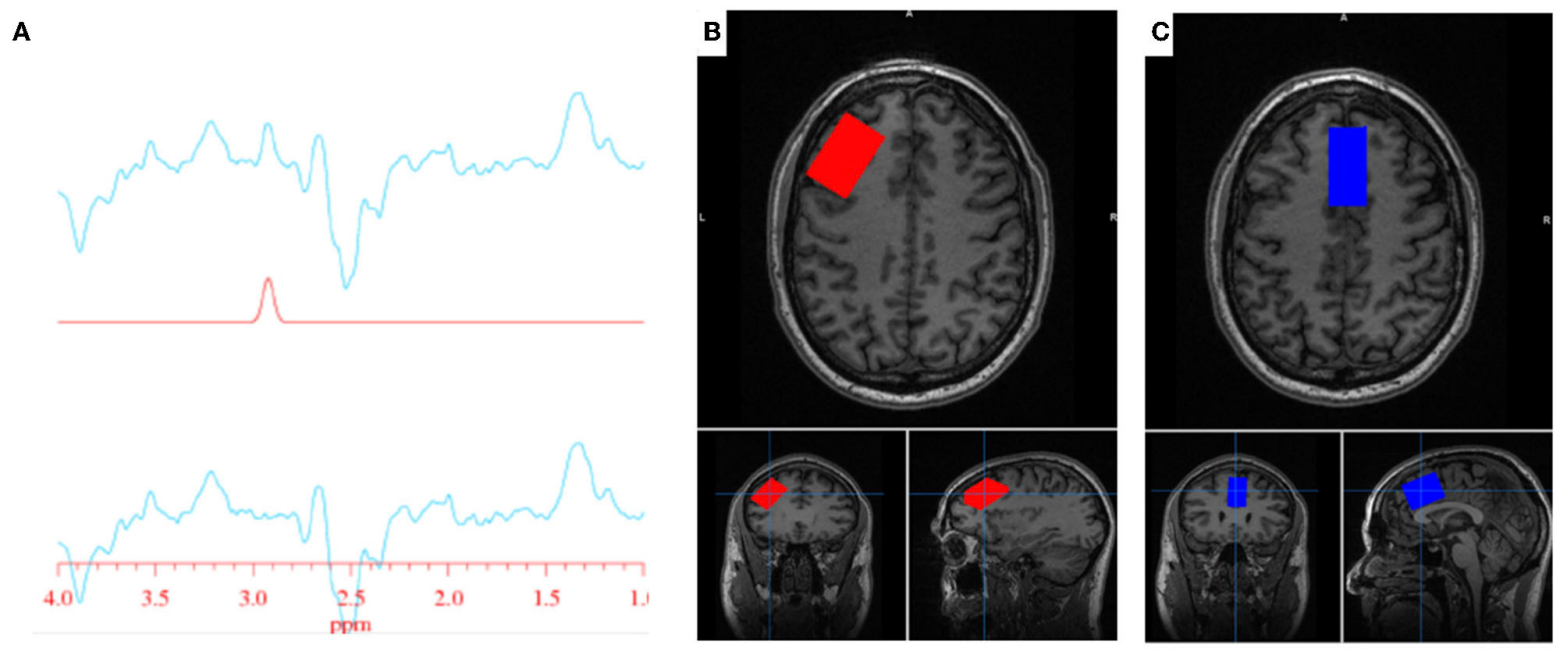
GSH metabolite acquisition. **(A)** Fitted GSH spectra obtained at 2.95 ppm (top line: difference spectrum; middle line: GSH model fit; bottom line: difference spectrum—model fit). **(B)** Voxel placement in the left DLPFC; **(C)** Voxel placement in the ACC.

All participants completed an MRS scan where spectra were obtained from one region of interest: the anterior cingulate cortex (ACC). A subset of participants also completed an MR scan where spectra were obtained from the ACC as well as the left DLPFC (Figure 1). Voxel dimensions for both ROIs were 4 cm x 2 cm x 3 cm, resulting in a nominal size of 24 cc. Shimming was performed using the manufacture automated shimming routine (AUTOSHIM), to achieve a full-width at half maximum (FWHM) ≤ 10 Hz. The MEGA-PRESS sequence was used to obtain MRS spectra as previously described (43, 44). MEGA-PRESS acquires spectra under two different conditions in an interleaved manner: editing “on,” which applies a frequency selective RF inversion (editing) pulse targeting the protons of GSH's cysteine moiety at 4.56 ppm; and editing “off” with editing pulse set to 7.5 ppm, a region with no metabolite resonances. Upon subtraction of the “on” and “off” conditions, the edited-GSH resonance at 2.95 ppm is observed, uncovered from the previously overlapped Cr resonant peak (Figure 1). Data acquisition parameters were: TE = 68 ms; TR = 1.5 s; spectral width = 5,000 Hz; number of points per spectrum = 4,096; NEX = 8; total averages acquired = 512; editing RF pulse width = 14.4 ms; scan time = 13:12 (min:sec).

IDL-based software [XsOs-NMR (45)] was used to process the edited GSH and the unsuppressed water spectra. Raw MRS data from each coil was combined in the time domain based on coil sensitivity (46) from the unsuppressed water signal, weighted by the sum of squares of the signal intensities from each coil. The data was spectrally apodised with a 3 Hz Gaussian filter and then zero filled to 8,192 points, prior to being Fourier transformed. Frequency alignment, additional manual phasing and baseline correction was performed on the data prior to fitting. Edited GSH and unsuppressed water peaks were modeled using pseudo-voight fitting functions and then fitted in the frequency domain using a highly optimized public-domain Levenberg-Marquardt non-linear least-squares minimization routine, MPFIT (47). Due to the manual phasing and baseline correction that require user input, the data set was randomized and processed two more times by the same user, resulting in three measurements per scan. The measurements were averaged together, and the standard deviation (SD) was calculated. The coefficient of variability (%CV = SD/average) was used to assess the reproducibility of the user. Histograms of the %CV could be used to identify outliers. We found that a %CV threshold of 10% yielded good results and excluded spectra that were visibly of poor quality. SPM12 (48) was used for tissue segmentation of the T1 images. MRS voxel and image registration and fractional tissue within voxel was performed using Gannet and SPM12 (49, 50); data were inspected for correct voxel placement.

### 2.4. Blood samples

Participants provided peripheral blood samples as part of a larger study (51). Venous blood was drawn into a 10-mL K2EDTA tube and left at room temperature for ~45 min before a 20-min centrifugation at room temperature. Plasma supernatant was then aliquoted and frozen at −80°C until analysis. Biofluid analysis comprised the quantitation of MMP-9, TIMP-1,2, and MPO using Simple Plex™ cartridges on the automated Ella^®^ fluorescence-based detection immunoassay system (ProteinSimple, Biotechne, San Jose, CA, USA) (52). Simple Plex cartridges were run according to the manufacturer's instructions, and data were processed automatically using default software settings as outlined in the Ella User's Guide. About an hour after test initiation, triplicate results for every analyte of each sample are provided and blood analyte concentrations are reported as the calculated mean of triplicate values.

### 2.5. Statistical analysis

Descriptive statistics (mean/median, standard deviation/interquartile ranges) were calculated for participant demographics and medical history (e.g., age, sex, race, and questionnaire scores). Group differences were evaluated by independent samples *t*-tests, Mann–Whitney *U* tests, or Chi square tests where appropriate. Independent samples *t*-tests were employed to evaluate group differences in GSH (in the ACC and DLPFC), MPO, MMP-9, and TIMP-1,2, with follow up tests completed to control for age, sex, BMI, and cannabis. Additional *t*-tests were completed in the PTSD only group to assess differences in GSH and blood markers between subgroups of PTSD participants (e.g., medication, history of brain injury, comorbid MDD). Two-tailed Pearson correlations were employed to evaluate possible correlations between (1) peripheral and central markers of oxidative stress and (2) markers of oxidative stress and PTSD characteristics and symptoms. Next, interacting variables between centered peripheral blood marker data and group status were computed (biomarker ^*^ group) and entered into a linear regression model to predict brain GSH in the ACC and DLPFC. All statistical analysis was conducted using IBM SPSS Statistics 27 (Armonk, New York, USA).

## 3. Results

### 3.1. Participants

Thirty-two participants with PTSD and 24 healthy controls (HC) were enrolled and scanned with MRS in the current study. ACC single-voxel MRS was acquired in 32 PTSD participants and 24 HC; of those 30 PTSD participants and 24 HC had usable data. DLPFC single-voxel MRS was acquired in 17 PTSD participants and 24 HC and of those 14 PTSD participants and 18 HC had usable data. Peripheral venous blood samples were obtained from 48 participants (*n* = 25 PTSD and *n* = 23 HC). One hundred percent of samples across all four blood biomarkers (MPO, MMP-9, TIMP-1, and TIMP-2) were within the level of detection and had a triplicate CV value < 20%. Of the PTSD participants with blood marker data, 23 had GSH scan data in the ACC and 14 in the DLPFC. Of the HC participants with blood marker data, 19 had GSH scan data in the ACC and 17 in the DLPFC (see Supplementary Figure 1 for sample visualization). A table of demographics for participants with available MRS data in the ACC is presented in Table 1. A separate table of demographics is available for participants with usable MRS data in the DLPFC (Supplementary Table 1). Participants with PTSD were older than HC participants and reported using more cannabis compared to HC.

**Table 1:** Participant demographics: PTSD and all healthy controls (ACC only).

	**PTSD (*n* = 30)**	**Healthy controls (*n* = 24)**	***P*-value**
Age, years	42.5 ± 10.5	33.5 ± 13.5	0.011
Sex, male *n* (%)	16 (53)	10 (50)	0.817
NIH race, Caucasian *n* (%)	24 (80)	10 (50)	0.103
BMI (kg/m^2^)	26.6 ± 3.7	25.1 ± 4.7	0.224
Years of education	16.4 ± 3.4	16.2 ± 2.6	0.834
Cigarette smokers, *n* (%)	4 (13)	1 (5)	0.87
Positive THC on MRI day, *n* (%)	10 (33)	3 (15)	0.148
Current cannabis use, *n* (%)	9 (30)	6 (30)	0.809
Cannabis (g)/week	13.3 ± 23.2	0.88 ± 1.2	0.492
Alcohol drinks/week	3.8 ± 5.4	2.9 ± 3.3	0.572
**Questionnaires**			
BDI, median (range)	18 (4–39)	4.5 (0–7)	< 0.001
GAD-7, median (range)	10.5 (0–21)	0.5 (0–5)	< 0.001
PHQ-9, median (range)	13 (1–27)	2.5 (0–4)	< 0.001
**Tissue fractions**			
**ACC**			
CSF fraction	0.236 ± 0.58	0.219 ± 0.67	0.348
WM + GM FRACTION	0.762 ± 0.59	0.779 ± 0.69	0.353
***DLPFC***			
CSF fraction	0.131 ± 0.038	0.119 ± 0.032	0.354
WM + GM fraction	0.865 ± 0.04	0.875 ± 0.034	0.445

Values are mean ± SD unless otherwise indicated.

ACC, Anterior Cingulate Cortex; BMI, Body Mass Index; BDI, Beck Depression Inventory; CSF, Cerebrospinal Fluid; DLPFC, Dorsolateral Prefrontal Cortex; GAD-7, General Anxiety Disorder-7; g, grams; NIH, National Institute of Health; PHQ-9, Patient Health Questionnaire-9; THC, Tetrahydrocannabinol.

PTSD specific characteristics are presented in Table 2. Participants scored ~36 on the PSS and 60 on the PCL questionnaires and had been suffering from PTSD related symptoms for 6 years. Twenty-five percent of participants were also diagnosed with current co-morbid MDD and half reported a history of mild traumatic brain injury (mTBI). The majority (88%) of participants were on medication, the most common type being selective serotonin reuptake inhibitor (SSRI)s, followed by cannabis (25%). Although the primary trauma was occupation related in 84% of participants, participants reported a range of trauma exposure including early childhood trauma (38%) and physical (56%) and sexual (22%) violence.

**Table 2:** PTSD characteristics.

	**PTSD in the ACC (*n* = 30)**	**PTSD in the DLPFC (*n* =1 4)**
PSS, median (range)	30 (11–51)	32 (11–48)
PCL, median (range)	59 (26–83)	59 (26–76)
Duration of PTSD (years)	7.2 ± 7.7	5.8 ± 4.1
Age of onset (years)	35.9 ± 10.2	34.7 ± 6.8
Current MDD, *n* (%)	7 (23)	5 (35)
History of mTBI, *n* (%)	15 (50)	6 (43)
**Medication use**, ***n*** **(%)**	**28 (93)**	**12 (85)**
Cannabis	8 (26)	7 (50)
SSRI	12 (40)	7 (50)
SARI	4 (13)	2 (14)
SNRI or NDRI	5 (16)	5 (35)
Atypical antipsychotics	2 (6)	1 (7)
Benzodiazepines	5 (17)	6 (43)
PDE5 inhibitor	1 (3)	1 (7)
Alpha blocker	1 (3)	1 (7)
Operation-related PTSD, *n* (%)	26 (86)	14 (100)
**Lifetime trauma exposure**, ***n*** **(%)**		
Natural disaster	10 (33)	5 (35)
Early childhood trauma	9 (30)	7 (50)
Physical violence	15 (50)	9 (64)
Sexual violence	6 (20)	3 (21)
Accident	18 (60)	10 (71)
War zone	9 (30)	7 (50)

Values are mean ± SD unless otherwise indicated.

MDD, Major Depressive Disorder; mTBI, Mild Traumatic Brain injury; NDRI, Norepinephrine-Dopamine Reuptake Inhibitors; PDE5, Phosphodiesterase Type 5; PSS, PTSD Symptom Scale; PCL, PTSD Checklist; SARI, Serotonin Antagonist and Reuptake Inhibitors; SNRI, Serotonin-Norepinephrine Reuptake Inhibitor; SSRI, Selective Serotonin Reuptake Inhibitor.

### 3.2. No group differences in brain levels of GSH

**Figure 2:**
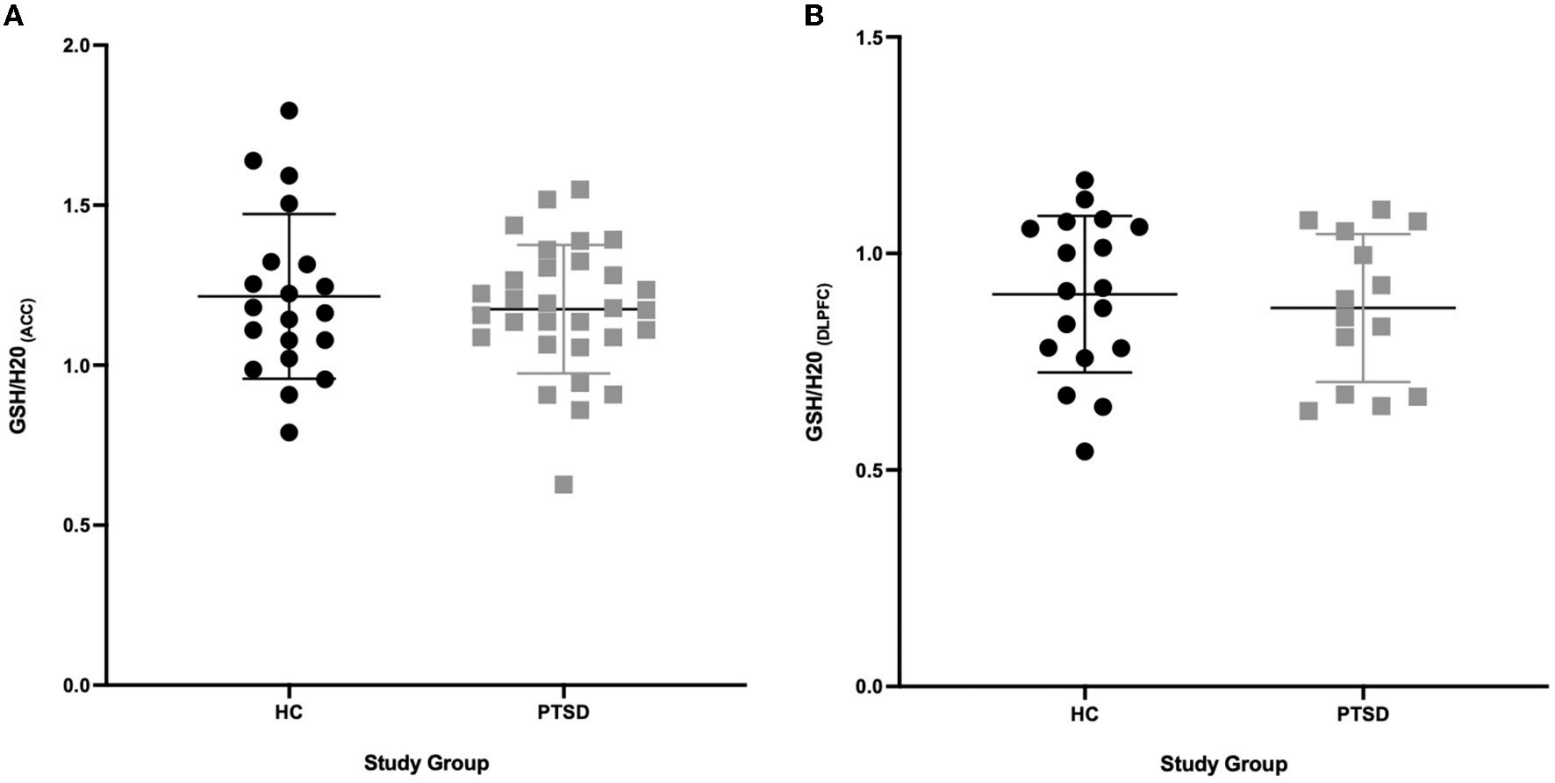
GSH between PTSD and HC in the ACC and DLPFC. **(A)** No difference (*P* = 0.536) in GSH concentrations in the ACC between PTSD participants (*n* = 30) and HC (*n* = 20). **(B)** No difference (*P* = 0.618) in GSH concentrations in the DLPFC between PTSD participants (*n* = 14) and HC (*n* = 18).

We found no differences in brain levels of GSH in the ACC (-3%, *p* = 0.536) or in the DLPFC in PTSD vs. HC (−3.5%, *p* = 0.618, see Figure 2). GSH levels were not related to age (ACC: *p* = 0.621; DLPFC: *p* = 0.805), sex (ACC: *p* = 0.681; DLPFC: *p* = 0.142) and BMI (ACC: *p* = 0.492; DLPFC: *p* = 0.527) in the overall sample and in the groups independently (PTSD and HC; *P* > 0.2). Testing positive for cannabis on scan day did not affect GSH levels in HC participants (4 THC+ vs. 20 THC-; ACC: *p* = 0.449; DLPFC: *p* = 0.326); however, GSH levels in the DLPFC were marginally higher in PTSD participants who tested positive for cannabis (*n* = 10) on scan day (*p* = 0.053, 25% higher), compared to those who tested negative (*n* = 4). Use of SSRIs did not influence brain levels of GSH (ACC *n* = 12/30: *p* = 0.275; DLPFC *n* = 7/14: *p* = 0.912). There was no difference in GSH levels in the ACC between PTSD participants with (*n* = 15) and without (*n* = 15) a history of mTBI (*p* = 0.449); however, GSH was 24% higher in the DLPFC (*p* = 0.03) in PTSD participants with a history of mTBI (*n* = 6) compared to those without (*n* = 8). Comorbid MDD (PTSD + MDD) did not affect GSH in the ACC (*p* = 0.623, *n* = 23 PTSD, *n* = 7 PTSD+MDD), nor in the DLPFC (*p* = 0.457, *n* = 9 PTSD, *n* = 5 PTSD + MDD).

### 3.3. Are there group differences in circulating blood markers?

There were no group differences in circulating plasma concentrations of MPO (*p* = 0.887), MMP-9 (*p* = 0.345), or TIMP-1 (*p* = 0.881). TIMP-2 was marginally lower in PTSD participants compared to HC (11%; *p* = 0.052). All group means are presented in Table 3.

**Table 3:** Group differences GSH and peripheral blood markers.

**Outcome**	**HC**	**PTSD**	***P*-value**
GSH_(acc)_	1.215 ± 0.257	1.175 ± 0.201	0.536
GSH_(dlpfc)_	0.906 ± 0.181	0.874 ± 0.171	0.618
MPO (pg/mL)	31,128 ± 17,241	30,506 ± 12,850	0.887
MMP-9 (pg/mL)	1,17,955 ± 56,328	133,455 ± 56,132	0.345
TIMP-1 (pg/mL)	87,731 ± 16,776	88,445 ± 16,100	0.881
TIMP-2 (pg/mL)	83,992 ± 18,725	75,096 ± 11,697	0.052

Data presented as mean ± SD.

ACC, Anterior Cingulate Cortex; DLPFC, Dorsolateral Prefrontal Cortex; HC, Healthy Control; GSH, Glutathione; MMP, Metalloproteinase; MPO, Myeloperoxidase; pg/mL, picograms per milliliter; PTSD, Post-traumatic stress disorder; TIMP, Tissue inhibitors of Metalloproteinase.

There was no effect of sex (*p* = 0.321; *p* = 0.362; *p* = 0.301; *p* = 0.216) and BMI (*p* = 0.564; *p* = 0.209; *p* = 0.405; *p* = 0.935) on MPO, MMP-9 and TIMP-1 and TIMP-2 concentrations in the sample overall. Age was positively related to TIMP-1 levels in the overall sample (*p* = 0.024). An analysis of variance taking age into consideration did not change the TIMP-1 finding (*p* = 0.463).

Testing positive for cannabis on scan day did not affect MMP-9, TIMP-1 and TIMP-2 concentrations (*P* > 0.4). However, PTSD participants testing positive for cannabis on scan day (*n* = 10) had nominally higher MPO concentrations (*p* = 0.167, 26% higher) compared to PTSD participants who tested negative (*n* = 15). Use of SSRIs did not appear to influence circulating concentrations of MPO (*p* = 0.522), MMP-9 (*p* = 0.645), nor TIMP-2 (*p* = 0.337). Concentrations of TIMP-1 were 15% higher (*p* = 0.074) among PTSD participants on SSRIs (*n* = 13) compared to participants not on SSRIs (*n* = 12). There were no differences in circulating concentrations of MMP-9 (*p* = 0.697), MPO (*p* = 0.544), TIMP-1 (*p* = 0.252), nor TIMP-2 (*p* = 0.971) between PTSD participants with (*n* = 13) and without (*n* = 12) a history of mTBI. PTSD + MDD (*n* = 18) and PTSD only (*n* = 7) participants did not differ in circulating levels of MMP-9 (*p* = 0.282), TIMP-1 (*p* = 0.394), nor TIMP-2 (*p* = 0.784). PTSD+MDD (*n* = 7) participants had 30% higher (*p* = 0.015) MPO concentrations compared to PTSD only participants (*n* = 18).

### 3.4. Relationship between GSH and peripheral blood markers

**Figure 3:**
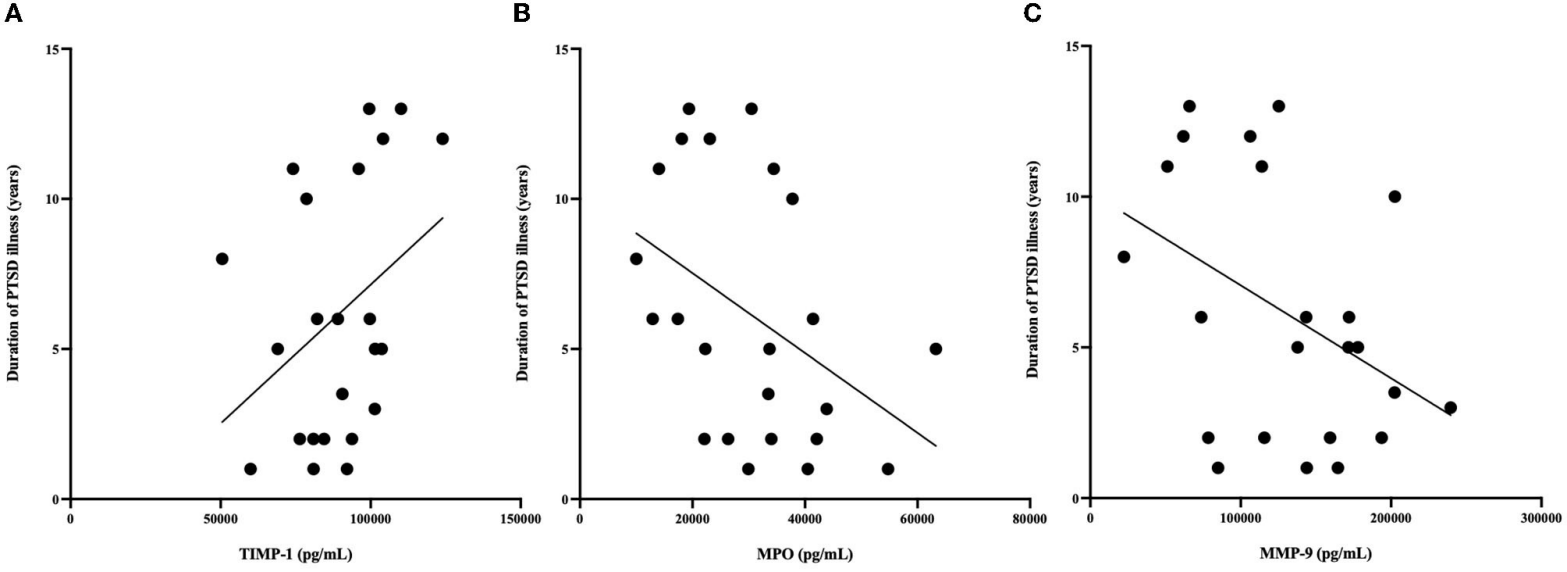
**(A)** Relationship between MPO (pg/mL) and duration of illness. MPO concentrations were negatively correlated with duration of PTSD illness (*R* = −0.566, *P* = 0.044). **(B)** Relationship between MMP-9 (pg/mL) and duration of illness. MMP-9 concentrations were negatively correlated with duration of PTSD illness (*R* = −0.41 *P* = 0.05). **(C)** Relationship between TIMP-1 (pg/mL) and duration of illness. TIMP-1 concentrations were correlated with duration of PTSD illness (*R* = 0.367, *P* = 0.089). *n = 23 since age at time of PTSD diagnosis was not available for 2 PTSD participants.

Correlational analysis was employed to evaluate relationships between central and peripheral markers of oxidative stress and PTSD clinical characteristics within the PTSD group. GSH in the ACC was positively correlated with circulating concentrations of TIMP-2 (*R* = 0.539, *p* = 0.008). MPO (*R* = −0.566, *p* = 0.044) and MMP-9 (*R* = −0.441, *p* = 0.05) were negatively correlated and TIMP-1 (*R* = 0.367, *p* = 0.089) was marginally positively correlated with duration of PTSD illness (see Figure 3). There were no other relationships between brain, peripheral markers of oxidative stress and symptom scores on questionnaires assessing PTSD, anxiety, nor depression (*P* > 0.3).

**Figure 4:**
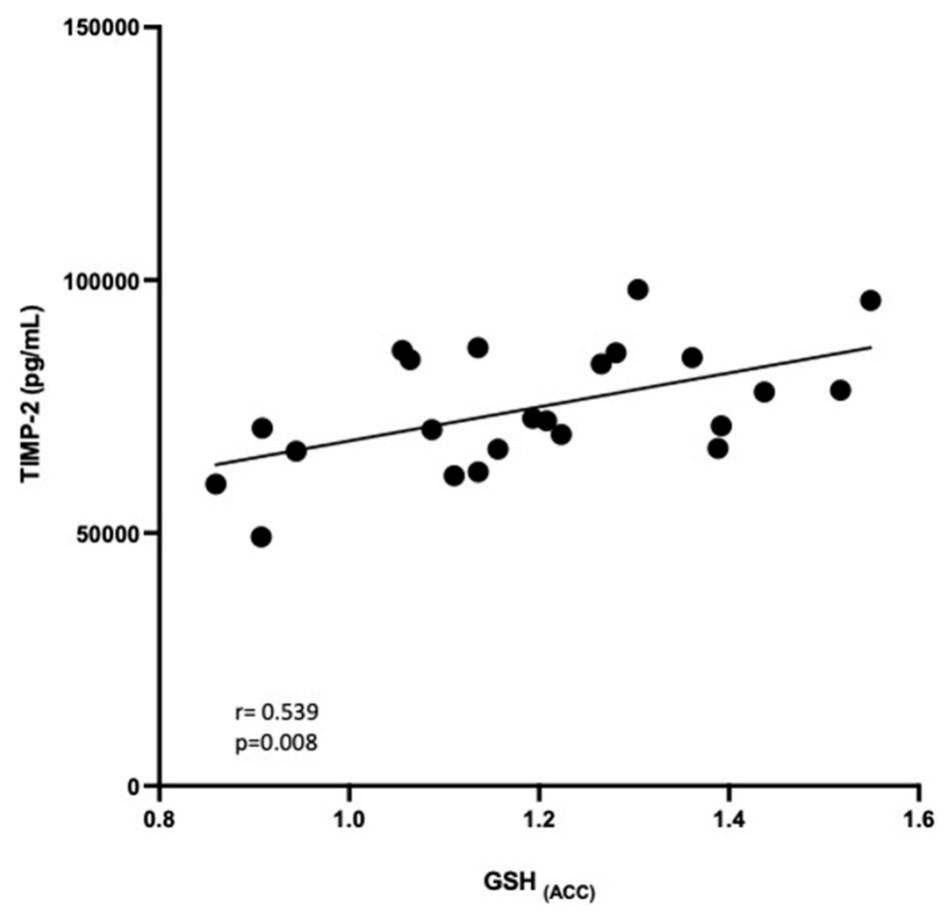
Relationship between GSH in the ACC and TIMP-2 concentrations (pg/mL). GSH in the ACC was positively correlated with TIMP-2 concentrations (*R* = 0.539, *P* = 0.008).

To determine if the relationship between peripheral markers and central GSH concentrations was group dependent, interaction terms were calculated for group ^*^ peripheral marker and entered into linear regression models to predict GSH in the ACC and DLPFC. There were no significant group^*^blood marker interactions in predicting GSH in the DLPFC (*P* > 0.7). Additionally, there were no group^*^blood marker interactions for MMP-9 nor TIMP-1 (*P* > 0.4). The relationship between TIMP-2 and GSH in the ACC seemed marginally group dependent (β = −0.784, *p* =0.051, see Figure 4), where the relationship was positive in PTSD participants but negative among HC participants. The relationship between MPO and GSH in the ACC also appeared to be marginally influenced by group (β = −0.389, *p* =0.099), where the relationship was positive among PTSD participants and negative among HC participants.

## 4. Discussion

To the extent of our knowledge, this is the first study to quantify brain GSH, using a validated MRS method (53), and explore relationships between peripheral MMPs, TIMPs, and MPO in participants with PTSD and HC. We did not observe any significant group differences in brain GSH or circulating concentrations of peripheral blood markers. Interestingly, peripheral TIMP-2 concentrations (which were marginally lower in PTSD) were positively correlated with GSH in the ACC within the PTSD group only. Additionally, we observed that, in PTSD participants, GSH in the DLPFC (but not in the ACC) is elevated in cannabis users and in individuals with a history of mTBI (albeit a small *n* = 10 sample size); that MPO was associated with cannabis use and comorbid MDD and that TIMP-1 was elevated among PTSD participants who endorsed SSRI use. Finally, duration of PTSD illness was negatively related with MMP-9 and MPO.

### 4.1. Group differences

Our null GSH finding in the ACC and DLPFC is at odds with Michels et al. (5) who reported a 22% increase in GSH (in ACC and DLPFC) among PTSD participants. Notably, the GSH acquisition methods were different between studies; while both employed MEGA-PRESS to acquire, Michels et al. (5)'s scanning protocol was optimized for detecting GABA and not GSH, while our scanning protocol was optimized for GSH. Next, PTSD participants enrolled in both studies were suffering from PTSD for similar durations (~5 years) and were similar ages (38 vs. 42 years). However, the current study's PTSD sample consisted of 16 males (53%) while Michels et al. (5)'s sample was 91% (11/12) female. Additionally, we enrolled 8 cannabis users while Michels et al. (5) did not report substance use. We observed marginally higher GSH (DLPFC) in PTSD participants who reported cannabis use (*n* = 10). Previous research has reported no difference in brain GSH (medial PFC) between healthy controls and regular cannabis users (54), while preclinical research has observed increased brain GSH following administration of cannabis (55). A recent review assessing the therapeutic potential for cannabis in counteracting inflammation and oxidative stress (56) concluded that while preclinical evidence supports this, the clinical evidence in humans is not convincing. We also observed nominally higher (16%) GSH (DLPFC) in PTSD males compared to females and higher GSH (DLPFC) among PTSD participants with a history of mTBI (*n* = 6). Previous research has associated increased brain GSH in a persistent concussion symptom cohort (57) and athletes exposed to repetitive head impacts during sport (58). The authors speculated that increased GSH reflected a compensatory response to ongoing inflammatory processes related to brain injury. That this finding was only observed in the DLPFC (and not the ACC) in our cohort is in line with research observing frontal cortical thinning in blast exposed veterans (59), decreased activity (60), and white matter damage (61) in the PFC following mTBI. It is possible the ongoing inflammatory injury processes related to mTBI exacerbated oxidative stress in this region.

There are several possible reasons why the current study did not observe group differences in brain GSH. First, GSH is present in low concentrations in the brain (62), therefore any changes related to oxidative damage that might occur in PTSD might not be reflected in GSH quantifications. Several studies report alterations in peripheral plasma concentrations of GSH and GSH related enzymes, however research suggests central GSH dysfunction differs from peripheral dysfunction (62). It is also possible that any oxidative damage that might occur in PTSD, is not sufficient to alter GSH concentrations. GSH is synthesized *de novo* in the brain by astrocytes and research has demonstrated increased GSH synthesis during oxidative stress-related toxicity *in vitro*, proposing *in vivo* synthesis of this antioxidant might be sufficient to not detect significant changes (14). Perhaps concentrations of the molecules from which GSH is derived might reveal group differences in this oxidative stress defense system. Follow-up investigations should continue to explore the role brain GSH and other central markers of oxidative stress might have in PTSD.

### 4.2. TIMP-2 is positively correlated with GSH in the ACC

Although we did not detect group differences in GSH between groups, we observed a positive relationship between GSH in the ACC and TIMP-2 concentrations in the PTSD group only (TIMP-2 was non-significantly, negatively correlated with GSH in the ACC in the HC group). Notably, TIMP-2 was marginally lower in PTSD compared to HC. This group dependent relationship of TIMP-2 and GSH suggests GSH could still be implicated in PTSD. Lower TIMP-2 has been reported in other psychiatric disorders, including MDD (63), and schizophrenia (64). Interestingly, TIMP-2 knock out mice show deficits in fear potentiated startle (31), a relevant feature of PTSD (65). TIMP-2 is a tissue inhibitor for MMP-2, another ECM protein. MMP-2 has been shown to have a role in synaptic plasticity (66) and be upregulated by noradrenaline (67)—two biological mechanisms implicated in PTSD. ROS can activate MMPs (including MMP-2) and simultaneously decrease concentrations of TIMPs (including TIMP-2) and this can contribute to BBB permeability (68). It is possible that ongoing oxidative stress in the brain related to PTSD is depleting GSH concentrations and the lower TIMP-2 is detected in peripheral circulation due to enhanced BBB permeability. Additionally, MMP-2 is frequently implicated in cardiac pathologies (69), a common outcome in those diagnosed with PTSD (70). Therefore, more research should explore the role TIMP-2 and MMP-2 may have in PTSD, and how they not only relate to central oxidative stress processes, but common somatic comorbidities in PTSD as well.

### 4.3. Relationships with duration of PTSD

We observed duration of PTSD illness (years) was negatively correlated with MPO and MMP-9 (after controlling for age). Michels et al. (5) reported a positive correlation between GSH in the DLPFC and duration of PTSD in their cohort. While we believe that this is the first study to observe this relationship in PTSD, MPO has been positively correlated with duration of bipolar disorder (71), and higher MMP-9 has been reported in younger youth diagnosed with bipolar disorder compared to older adults (72). MPO is an enzyme that carries out peroxidative activities and is released by neutrophils, also reflecting the state of neutrophils in the innate immune response (73). Research has also reported increased MPO in MDD (33), neurodegenerative disorders including Alzheimer's Disease (74), and preclinical PTSD (32). It is noteworthy that we observed lower MPO concentrations in participants who had been suffering from PTSD for longer. There was no difference in severity of PTSD or symptom scores between participants diagnosed with PTSD recently or several years prior. During the innate immune response, neutrophils respond by releasing a burst of ROS (and MPO); however, research has suggested chronic stress can compromise this function (75). Therefore, it is possible the chronic duration of PTSD has resulted in a similar immune exhaustion in our cohort. MMP-9 is an ECM protein that has been increasingly implicated in psychiatric disorders, including PTSD (28). There is a strong line of research implicating MMP-9 in sleep and memory consolidation processes and MMP-9 is upregulated during contextual fear learning (76). Furthermore, treatment with an MMP inhibitor can disrupt fear reconsolidation (77) and fear memory (78) in preclinical research. Again, the negative relationship between duration of PTSD and MMP-9 is important, considering the research discussed above. Glucocorticoids, including cortisol, can regulate MMP-9 (79), therefore, it is possible that this negative relationship is related to chronic cortisol dysregulation in PTSD. MMP-9 is inhibited by TIMP-1, an MMP tissue inhibitor. TIMP-1 was positively related with duration of PTSD (before controlling for age). Preclinical research suggests TIMP-1 is implicated in fear and memory processes (80), and has shown to have a protective role in neurodegenerative disorder (81). Further research is required to understand the role MMPs, TIMPs, and MPO have in the chronic, progressive nature of PTSD (82).

### 4.4. Limitations

While this research is an important contribution to understanding oxidative stress in PTSD, it is not without limitations. First, we used a manual software (XsOs) to process (baseline correct and phase) our GSH spectra (manual processing yielded higher quality spectra than automatic processing software); we attempted to negate any operator bias by randomizing the data and having each data point processed three times by the same research staff member (SEW). Next, while the MEGA-PRESS sequence was optimized for GSH, the obtained signal is relatively small and can only be quantified with relatively large error. However, test re-test %CV of our GSH acquisition was 15%, which is within range of previous research (83, 84). Research (53) also demonstrated MEGA-PRESS is able to accurately quantify GSH at low physiological concentrations. Next, we had a small sample size (*n* = 14) in the DLPFC which made it difficult to make comparisons between regions (ACC vs. DLPFC) and dissect the influence of medication, mTBI, and cannabis on GSH in the region, therefore findings in this region should be interpreted with caution and merit further investigation. Additionally, it would have been useful to assess GSH in other regions implicated in PTSD, including the hippocampus and amygdala. Finally, while we believe the collection of peripheral blood markers complimented our central measures, it would have been informative to measure additional redox proteins directly implicated in oxidative stress processes, including peripheral TBARS and MDA concentrations.

### 4.5. Conclusions and next steps

In summary, the current study did not observe altered brain GSH concentrations in PTSD. We did however report a non-significant decrease in TIMP-2 in PTSD and TIMP-2 was positively correlated with GSH in the ACC in the PTSD group only. Finally, we observed negative relationships between MPO, MMP-9 and duration of PTSD illness. Our interesting findings among the peripheral blood markers warrant further investigation to understand how, if at all, systemic dysregulation of systems is implicated in the progression of PTSD over time. Future research is needed to better understand ongoing central oxidative stress processes in relation to peripheral mechanisms, and how these mediators change across the course of disease in people with PTSD.

## Data availability statement

The raw data supporting the conclusions of this article will be made available by the authors, without undue reservation.

## Ethics statement

The studies involving human participants were reviewed and approved by CAMH Research Ethics Board. The patients/participants provided their written informed consent to participate in this study.

## Author contributions

SR, JR, SK, and IB contributed to study conceptualization and study design. SW, JW, DG, TM, PT, and SC contributed to data collection and data processing. SW, SR, and IB contributed to manuscript drafting. All authors contributed to manuscript editing and reviewing.
