# Supplementary material for: Exploring brain glutathione and peripheral blood markers in posttraumatic stress disorder: a combined [1H]MRS and peripheral blood study
Source: Front Psychiatry. 2023 Jun 2;14:1195012. doi: 10.3389/fpsyt.2023.1195012 (PMC10272391; doi:10.3389/fpsyt.2023.1195012)
Supplement: Supplementary file 1 [file Table_1.DOCX]

Supplementary Material

Exploring brain Glutathione and peripheral blood markers in Posttraumatic stress disorder: a combined [1H]MRS and peripheral blood study

**Sarah E. Watling, MSc^1,2^ Shawn G. Rhind, Ph.D^3,4^ , Jerry Warsh, MD, Ph.D^1,2 5,6,7^ , Duncan Green^1,2^, Tina McCluskey, MSc^2,5^, Junchao Tong, Ph.D^2,5,6^, Peter Truong, Meng^2^, Sofia Chavez, PhD^2^ , J. Don Richardson, MD ^6,7,8,9^, Stephen J Kish, Ph.D^1,2,5,6,7^, Isabelle Boileau*, Ph.D^1,2,5,6^**

*** Correspondence:** Corresponding Author: isabelle.boileau@camh.ca

# Supplementary Figures and Tables

## Supplementary Figures

##
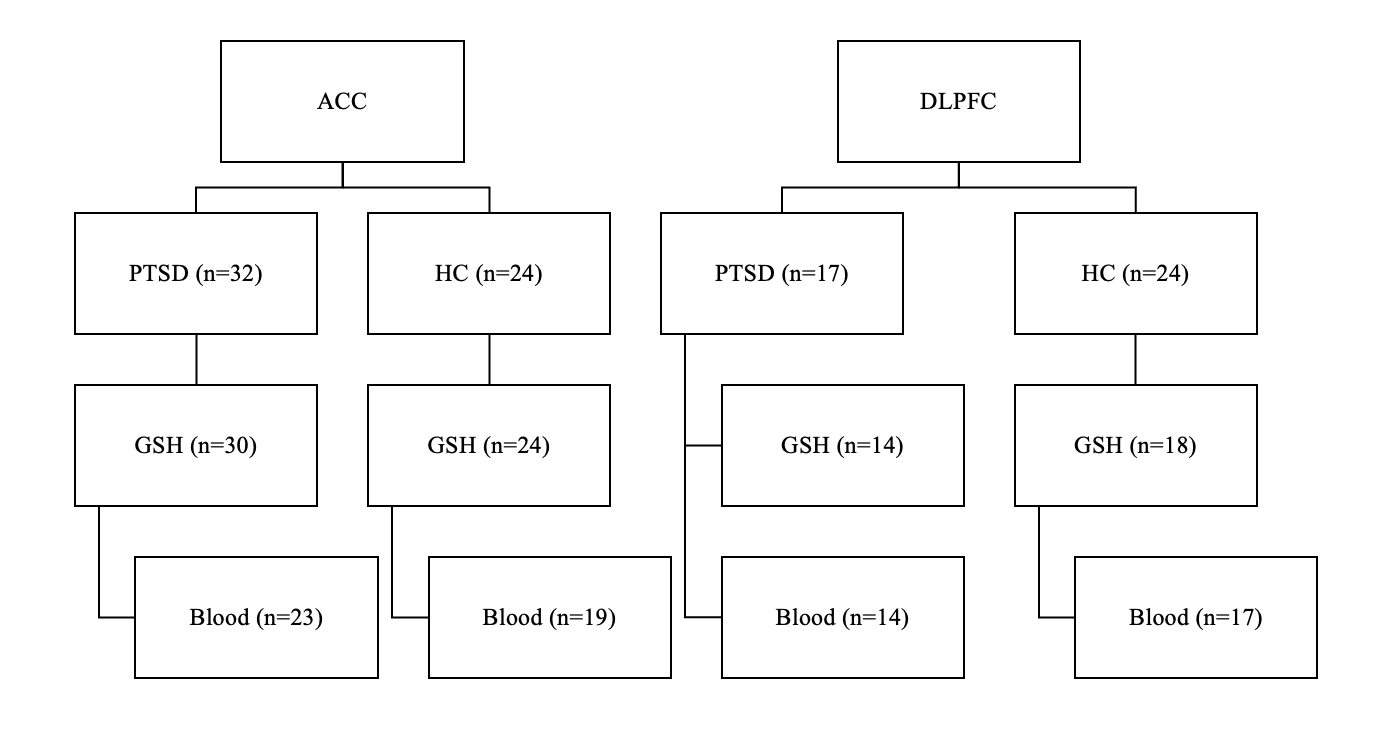


**Supplementary Figure 1**: Overview of data collection in study sample.

## Supplementary Tables

| Table S1: Participant Demographics: PTSD and all Healthy Controls (DLPFC only) | | | |
| --- | --- | --- | --- |
|  | PTSD  (n=14) | Healthy Controls (n=18) | p value |
| Age, years | 41.2 ± 8.4 | 33.5 ± 13.4 | 0.054 |
| Sex, male n(%) | 9 (64) | 7 (39) | 0.154 |
| NIH race, Caucasian n(%) | 24 (75) | 14 (58) | 0.344 |
| BMI (kg/m^2^) | 26.9 ± 4.5 | 25.1 ± 5 | 0.301 |
| Years of education | 15.1 ± 2.6 | 16.1 ± 2.8 | 0.289 |
| Cigarette Smokers, n(%) | 1 (3) | 1 (5) | 0.371 |
| Positive THC on MRI day, n(%) | 10 (71) | 4 (22) | 0.005 |
| Current Cannabis Use, n(%) | 8 (57) | 7 (39) | 0.361 |
| Cannabis (g) / week | 13.3 ± 23.2 | 1.4 ± 1.3 | 0.223 |
| Alcohol drinks / week | 3.2 ± 5.9 | 1.9 ± 1.9 | 0.535 |
| *Questionnaires* |  |  |  |
| BDI, median (range) | 21 (4-39) | 5 (0-16) | <0.001 |
| GAD-7, median (range) | 11 (0-21) | 1 (0-12) | <0.001 |
| PHQ-9, median (range) | 14 (1-27) | 2 (0-11) | <0.001 |
| *Tissue Fractions* |  |  |  |
| *ACC* |  |  |  |
| CSF Fraction | 0.236 ± 0.58 | 0.219 ± 0.67 | 0.348 |
| WM + GM Fraction | 0.762 ± 0.59 | 0.779 ± 0.69 | 0.353 |
| *DLPFC* |  |  |  |
| CSF Fraction | 0.131 ± 0.038 | 0.119 ± 0.032 | 0.354 |
| WM + GM Fraction | 0.865 ± 0.04 | 0.875 ± 0.034 | 0.445 |
| Values are mean +/- SD unless otherwise indicated  Anterior Cingulate Cortex (ACC); Body Mass Index (BMI); Beck Depression Inventory (BDI); Cerebrospinal Fluid (CSF); Dorsolateral Prefrontal Cortex (DLPFC); General Anxiety Disorder (GAD)-7; grams (g); National Institute of Health (NIH); Patient Health Questionnaire (PHQ)-9; Tetrahydrocannabinol (THC); | | | |

**
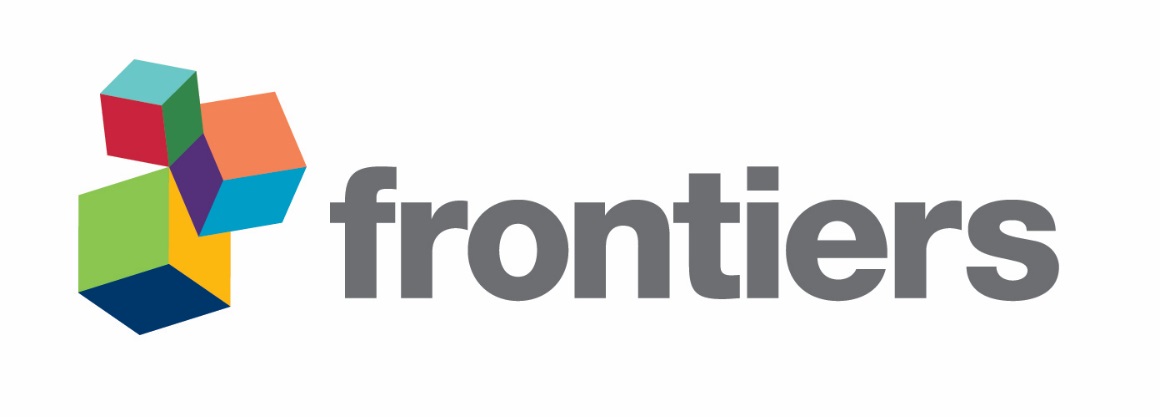
**
